# The silent HIV epidemic among pregnant women within rural Northern Tanzania

**DOI:** 10.1186/1471-2458-6-109

**Published:** 2006-04-27

**Authors:** Khadija I Yahya-Malima, Bjørg E Olsen, Mecky I Matee, Knut Fylkesnes

**Affiliations:** 1 Centre for International Health, University of Bergen, Bergen, Norway; 2 School of Nursing, Muhimbili University College of Health Sciences, Dar es Salaam, Tanzania; 3 Department of Training, Ministry of Health, Dar es Salaam, Tanzania; 4 Haydom Lutheran Hospital, Mbulu District, Manyara, Tanzania; 5 Department of Microbiology and Immunology, Muhimbili University College of Health Sciences, Dar Es Salaam, Tanzania

## Abstract

**Background:**

Many national antenatal clinics (ANC) based HIV surveillance systems in sub-Saharan Africa have limited coverage of remote rural sites, a weakness that compromises adequate estimation, monitoring and development of effective preventive and care programmes. To address this void in rural Manyara and Singida within Northern Tanzania, we conducted antenatal clinic-based sentinel surveillance.

**Methods:**

We consecutively enrolled 1377 counselled and consenting pregnant women attending ANC clinics for the first time during the current pregnancy. The study was conducted in six antenatal clinics, within three divisions of rural Manyara and Singida regions in 2003/2004. Interviews were conducted and blood samples for routine purposes were collected and tested for anti-HIV IgG antibody anonymously, using Bionor HIV-1 & 2 assay ^®^.

**Results:**

Among enrolees, 94% (1296/1377) participated fully. The overall prevalence of HIV was 2.0% (95%CI: 1.34–2.97). The highest HIV prevalence was among women aged between 15–19 years in both rural and remote rural populations. The odds of HIV infection was 4.3 (95%CI: 1.42–12.77) times among women reporting more than one lifetime sexual partners compared with those with one partner. HIV infection was associated with history of genital sores or foul smelling discharge, OR 6.8 (95%CI: 2.78–16.66) and age at first pregnancy (2.5 times higher likelihood of infection if before the age of 18 years versus at a later age).

**Conclusion:**

Including rural remote sites, as part of the national ANC routine surveillance, is crucial in order to discover imminent silent epidemics such as the one described in this paper. Scaling up HIV prevention efforts is mandatory to prevent the imminent escalation of the HIV epidemic highly associated with a history of sexually transmitted infections (STIs), multiple sexual partners and pregnancies at a younger age. Ignorance of relevant knowledge and low utilisation of condoms underscores the urgency for large-scale preventive efforts. Research to capture a wider representation of the risk factors in the general population should be a priority to enable further customised HIV prevention efforts.

## Background

Surveillance of human immunodeficiency virus (HIV) infection among pregnant women attending antenatal care clinics (ANC) has been the mainstay system of monitoring of HIV epidemic in most countries of sub-Saharan Africa [1-5]. Several studies from the 1990s showed that ANC based prevalence estimates approximated prevalence levels in the population of men and women aged 15–49 years [6-10]. However, the validity of prevalence estimates based on national ANC surveillance systems will depend on how accurately the selected sentinel sites represent the whole population [11]. In many resource-constrained countries, the quality of surveillance had been questionable for various reasons. It is common to find an uneven distribution of sentinel surveillance favouring sites mostly in urban and semi-urban areas [12,13]. This has led to limited surveillance information from rural areas, especially those that are inaccessible due to difficult terrain and lack of roads, and where literacy levels are generally low. These scenarios complicate attempts to determine the real magnitude and trends of HIV infection in different population groups, and make it difficult to develop appropriate preventive and control measures.

Very difficult terrain, poor roads and infrastructure, limited health facilities, and low literacy and socio-economic levels characterise the rural areas of Manyara and Singida regions within northern Tanzania. Since 1955, one of the main health providers of these rural settings has been the Haydom Lutheran Hospital (HLH), which is a 400- bed hospital owned by the Evangelical Lutheran Church of Tanzania (ELCT). The hospital works closely with national, regional and local government and health authorities, and offers its services within the national health plan, using national guidelines.

In September 2002, the Haydom Lutheran Hospital, with funding from the Royal Norwegian Embassy in Dar es Salaam, Tanzania, initiated a multi-sectoral collaboration for a comprehensive HIV prevention-intervention program. The project aimed to reduce the risk of HIV transmission; vulnerability of HIV infected persons and impact of HIV in the catchment area [14].

Generating local knowledge on the epidemiological context of HIV infection is of critical importance to plan interventions in the area. We present results from the antenatal clinic-based sentinel surveillance from various rural sites in Manyara and Singida regions in 2003/2004.

## Methods

### Study area

We conducted this study from November 2003 to April 2004 within the catchment area of the Haydom Lutheran Hospital (HLH), located in Mbulu district of Manyara region within Northern Tanzania. The area constitutes three administrative divisions in the rural areas of two regions of Manyara and Singida. In Manyara region, we included two divisions, namely Dongobesh division from Mbulu district and Basotu division from Hanang district. From Singida region, Nduguti division from Iramba district was included in the study. Based on the 2002 census [15], with extrapolation to 2003 of 3.8% annual growth for Manyara Region and 2.3% annual growth for Singida region, the three rural divisions had a total population of 259,292 persons.

In the study area, all four main language groups of East Africa are represented. The Iraqw (Cushitic), Datoga (Nilotic), and Iramba (Bantu) are the largest groups, although the commonly spoken language is Kiswahili.

The main economic activities in the area are subsistence farming and pastoralism. The HLH provides the main hospital level health care in the area.

### Sampling

Geographical stratification of the area into two strata (remote rural and rural) for each division (smaller units of a district) enabled us to conveniently sample two sentinel sites from each division, one from remote rural and one rural site. Six sentinel sites (three from remote rural and three from rural) within the three divisions were included in the study. The three rural clinics were the HLH clinic, the Nduguti Dispensary (government) and the Basotu Dispensary (government). From the remote rural areas, we had two HLH owned outreach clinics from Muslur (Dongobesh division) and Dangayda (Basotu division) and the Roman Catholic owned Mwanga Dispensary (Nduguti division). The selected sentinel sites provided adequate sample size and representative pregnant women within the catchment area based on sample size estimates [16]. We defined a "rural" residential area in this study as villages within the catchment area with more than two general utility shops and an established health facility. While "remote rural" villages are those found in the remote difficult terrain. Usually, these remote areas lack established health facilities or utility shops. Residents from remote rural areas have to travel by foot at least an average of 3 hours to get basic health services and at least 7 hours to the nearest hospital [17].

### Sample size

The sample size was estimate based on Statcal calculation of Epi Info computer package for descriptive studies version 6 [18]. Using the hospital based population prevalence of 2% [14], an estimate of 1000 pregnant women was considered adequate to detect the prevalence of HIV. We used the expected prevalence of 2% and worst acceptable prevalence of 0.98% at 95% confidence interval width of 1–3 [11]. Each of the six clinics was supposed to enrol a minimum of 167 pregnant women.

### Participants

The criteria for selecting pregnant women included all pregnant women aged between 15–49 years attending the ANC clinic for the first time during the current pregnancy during the surveillance period [11]. Routine investigations for first time visits in these clinics, necessitates blood withdrawal. The exclusion criteria included pregnant women who had previously visited the clinic during the surveillance period, and those aged less than 15 years or over 49 years. Voluntary counselling and testing (VCT) for HIV among pregnant women has been routinely integrated into the reproductive and child health (RCH) clinics as part of the prevention of mother to child transmission (PMTCT) of HIV [19]. This routine is for hospitals designated to provide anti-retroviral treatment. We consecutively enrolled all counselled and consenting pregnant women attending antenatal clinics for the first time during the current pregnancy within the catchment area from November 2003 to April 2004.

### Ethical issues and investigations

Both the Norwegian National Committee for Medical Research Ethics and the Tanzanian based National Institute for Medical Research (NIMR) gave ethical approval. Respective health and government authorities at regional and district level received copies of the ethical approval, as required for the implementation of the study. At the individual level, an oral informed consent was sought after detailed explanation of the purpose and benefits of surveillance for participation in the research only. It seemed necessary to reveal the establishment of the PMTCT unit at the hospital and its potential benefits unrelated to refusal or participation in the research.

### Data collection tools and testing

Questionnaires used for interviews had been translated into Swahili and translated back to English again to confirm their meaning. Information collected included basic demographic details and knowledge on HIV modes of transmission. Respondents were asked to agree or disagree with 10 statements regarding HIV related knowledge based on the protective effect of condoms, VCT and PMTCT, including main modes of infection and risky behaviour. An index was developed to sum up the scores of each statement, thus each correct response scored "one" and the highest score was 10. It was necessary and functional to collaborate with the hospital PMTCT team. The PMTCT team performed counselling and registered names with codes. They provided the codes for questionnaires and kept the identifiers from the research team for research purposes. The research team conducted interviews to consenting and counselled women using the codes. Screening for anti-HIV IgG antibody was performed anonymously for all blood samples collected for routine syphilis testing using Bionor HIV-1 & 2 assay^® ^(BIONOR AS, Skien, Norway). Ice coolers were used to pack blood collected at ANC clinics and transported to the HLH laboratory for testing. Bionor HIV 1& 2 assay ^® ^confirmatory test was used to confirm all samples that tested positive on screening. We linked the results of the test back to questionnaires using codes, but identities of individuals remained anonymous to the research team. Positively confirmed results by codes were submitted to the PMTCT team, which proceeded with the required procedures for PMTCT as required by the national HIV treatment guidelines [19].

### Statistical analysis

Statistical analysis utilised STATA 8.0 [20]. Chi-square test was used to test bivariate associations for categorical associations of HIV infection and other associated factors. Where appropriate, we used Fisher's exact test. A logistic regression model was used to explore the association of socio-demographic and other variables with HIV infection.

Variables with p-values < 0.25 on univariate analysis and after assessment of co-linearity were included in the multivariable model [21]. Variables included in the full multivariable model that were not significant were dropped after assessment of interaction. We did not find any interaction terms and the final reduced model was adjusted for age and residential area. These were history of genital sores and discharge, contraceptive use, number of sexual partners, age at first pregnancy, ever VCT and ever-used condom. We present odds ratios (OR), and 95% confidence interval (CI) for factors associated with HIV infection.

## Results

By the end of the surveillance period, 1377 pregnant women aged between 15 to 49 years were included. Of these, 41% were from remote hard to reach areas and the rest from other rural stratum. Among those enrolled, 4% (58/1377) refused to give blood for syphilis testing despite counselling and 2% (25/1377) of the blood samples were haemolysed before testing resulting in a participation of 94% (1296/1377). Examination of socio-demographic characteristics did not disclose significant differences between the participants and those who refused HIV testing.

**Figure 1:**
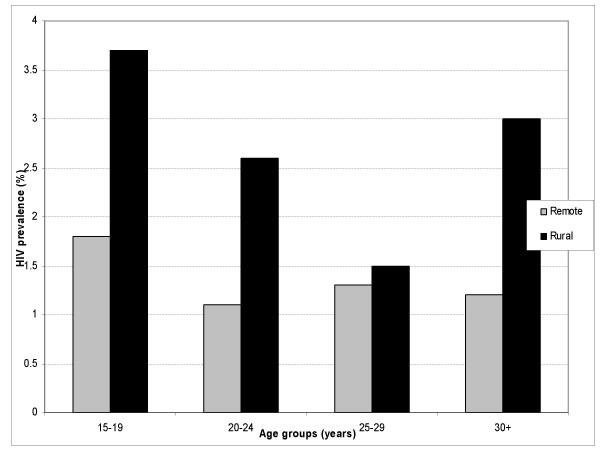
Age – specific HIV prevalence among antenatal attendees in Rural Manyara and Singida regions, Tanzania, 2003–2004.

The mean age of the studied population was 26.3 (SD ± 6.09) Table 1 presents general characteristics of the study population. The prevalence of HIV was 2.0% (95%CI: 1.24–2.76). Pregnant women residing in the rural areas were twice as likely to be HIV positive compared with those from remote rural areas; OR 2.0 (95% CI: 0.85–4.90). HIV prevalence was relatively higher at all ages in rural areas compared to remote areas (see Figure 1) The age group 15–24 years constituted 50% of those infected and HIV prevalence tended to be negatively associated with age.

We found no statistically significant association between HIV infection and level of education, but the prevalence tended to be higher among the group with no education compared with those educated. Regarding HIV related knowledge, 88.8% (n = 1151) of the study population scored below five on the index, and considered to lack adequate knowledge on the general modes of HIV transmission and prevention. The proportions of this knowledge deficit are similar for both areas, remote rural and rural. However, this lack of knowledge increased the likelihood of HIV infection 1 and a half times more OR 1.5 (95%CI: 0.49–4.29), though this was not statistically significant.

### Factors associated with HIV infection on univariate analysis

On univariate analysis, HIV infection was significantly associated with the number of lifetime husbands/sexual partners; OR 4.8 (95%CI: 1.75–13.17) and polygynous relationships (Fisher's exact test, p < 0.01) that characterise the majority of relationships 99% (1289/1296). Women who reported more than one lifetime sexual partner, had an HIV prevalence of 7.7% and had 4.8 times likelihood of being HIV infected, compared to those reporting one partner only. Women who had been using contraceptives of any kind, were more likely to be HIV infected; OR 2.8 (95%CI: 1.25–6.48) compared to non-users (Table 2). HIV infection was highly associated with first pregnancies at ages below 18 years compared to first pregnancies at ages above 18 years. A history of either genital sore or foul smelling genital discharge increased the odds of HIV infection by 4.6; OR 4.6 (95%CI: 2.06–10.39). Women, who had ever received voluntary counselling and testing, were twice more likely to be HIV infected; OR 2.6 (95%CI: 1.20–5.72). Single, divorced or separated women had 1.9 odds of being HIV infected but not significantly; OR 1.9 (95%CI: 0.65–5.72). Women who had ever used a condom were more likely to be HIV infected, OR 3.7 (95%CI: 1.25–11.18) compared to those who have never used condoms.

### Factors associated with HIV infection on multivariate analysis

HIV remained significantly associated with more than one of lifetime sexual partners OR 4.3 (95%CI: 1.42–12.77) and history of genital discharge or sore, OR 6.8 (95%CI: 2.78–16.66). In addition, women who had their first pregnancy at 18 years or older were less likely to be HIV infected compared to those being pregnant for the first time at less than 18 years OR 0.3 (95%CI: 0.13–0.89).

## Discussion

This study provides a comprehensive sentinel surveillance of HIV in these very remote rural regions of Manyara and Singida in Northern Tanzania. The HIV prevalence for the area was 2.0% with a rural: remote rural ratio of two. Compared to other rural areas in Tanzania, this prevalence is relatively low. For example in Mbeya region, the ANC-based prevalence in 2002 was 13.5% and 23.3% in Isoko and Itete, both being rural areas respectively, whereas in rural Moshi it was 16.6% [22]. However, there were signs indicating that the HIV incidence might be increasing in the area. First, epidemics on the increase will tend to show a negative association between age and infection. A second indication was the findings from hospital-based screening of HIV infection done in the main antenatal clinics in the same region in 1995/96 and 1999. These showed a prevalence of 0.3% [(95%CI: 0.04–1.1), (n = 2/733)] and 0.4% [95%CI: 0.07–1.7), (n = 2/467)], respectively [23]. A reflection of the relatively general low exposure to sources of infection rather than absence of risks factors that are also evident elsewhere [24]. This area had limited accessibility in the last decade, due to poor infrastructure, hence less traffic and reduced mobility [23]. This might have been among the factors that limited the sources and spread of infection. However, the situation is now changing with improvements in roads, increase in trade and mobility, which in turn may escalate the spread of HIV infection in these communities [25].

It is likely that the rural area with a relatively higher HIV prevalence presents marked current changes compared to the remote area.

This study included all pregnant women from all divisions that fall within the geographical boundaries of the catchment area. Though documented that in Tanzania, that 98% of pregnant women receive antenatal care [26], some areas lacked established clinics. In order to address this void, planned monthly outreach reproductive and child health (RCH) clinics from HLH and government RCH clinics take place throughout the year. Thus, biases due to non- attendance are likely to be minimal, owing to the placement and coverage of clinics, and absence of private clinics in the area. However, as these clinics are also entry points for PMTCT, VCT is mandatory in order to be tested, and this introduces potential selection bias since it requires consent. This might over-estimate the HIV prevalence among pregnant women since pregnant women perceiving themselves to be HIV infected from neighbouring areas outside the catchment area, might seek health services from this locality to benefit from the PMTCT intervention. Under-estimation is also a possibility since such women might also avoid such clinics for fear of confirming their HIV status regardless of the benefits. Nevertheless, recent studies have shown it is possible to estimate HIV prevalence data from PMTCT sites [27-29] with maximum coverage of clinics within localised areas, albeit with caution to the inherent biases.

Sample size estimates used hospital based HIV prevalence as a proxy, which might not be appropriate for subgroup analysis. However, this provided the best recent proxy estimate because the only previous ANC estimate was five years before the present study. In addition, the assumption was that "unlinked ANC HIV prevalence data" might represent the general population. Since the HLH is the main health care provider at hospital level in the area, HIV prevalence at hospital level might reflect the general population prevalence [30].

The lower number of the dependent variable, HIV positive, might have compromised the study findings in relation to the risks associated with HIV. In this respect, the associated risk factors were subject to random error, as reflected by the large confidence intervals around the point estimates. Thus, they reduced the ability to obtain significant statistical results with some of the variables.

We cannot assume that the findings of this study can be generalised to other rural areas elsewhere. Though the study represents a rural setting, where the majority of the population is less educated, with no stable income, cultural and traditional customs prevail.

The estimated prevalence might reflect the prevalence in the general population within the catchment area, as shown by validations of the ANC-based and population-based estimates [6,9,10]. These validations have been showing an overestimation of the prevalence among younger women and underestimation in older age group. Nevertheless, the HIV epidemic has been shown to be heterogeneous in terms of intensity, pace and impact within sub-regions [31]. Similar patterns may be found within different areas of the same country (8, 22, 31). Thus, local monitoring may reveal inherent patterns that can be optimised for preventive strategies.

There have been mixed conclusions on the risk of HIV with educational attainment, but most studies from sub-Saharan Africa have shown increased schooling to be associated with increased risk of HIV [7,9,32]. Studies that are more recent however, have indicated that this association changed direction in the 1990s. In young people, the likelihood of infection was higher in those with little education compared to those with more education [7,9,32,33]. The HIV prevalence tended to decline with educational attainment in our data. Women with an education of more than seven years have lower parity compared to those with less years of schooling in this setting, confirming less number of children with higher educational attainment, but not with increased odds of HIV infection.

The effect of reduced fertility because of HIV in an area of low prevalence is likely to be minimal since these are recent infections. The situation might change with time as the epidemic matures in this setting. However, its direction is uncertain, as educational data from antenatal clinics are prone to bias due to attendance influence by fertility and educational attainment. The general deficit in HIV related knowledge is not surprising since HIV related preventive services in the local area were introduced just recently as part of the larger preventive programme.

History of either genital sores or foul smelling genital discharge was found to be significantly associated with HIV in this study, OR 6.8 (95%CI: 2.78–16.66). This is in accordance with other studies [34,35]. Prevalent STIs enhance HIV transmission by increasing susceptibility through compromising mucosal integrity [35]. We are inclined to consider high prevalence of ulcerative STIs such as chancroid, syphilis and herpes simplex virus 2 [35-37], due to their greater disruption of genital mucosa. The effects of STI control have been suggested to be beneficial at the beginning of an HIV epidemic in other studies [38,39]. This is a strategy also applied in this local setting to prevent the spread of HIV

Women who reported more than one lifetime sexual partner had four times likelihood of being HIV positive, OR 4.3 (95%CI: 1.42–12.77). Few women (5%) report more than one lifetime sexual partner. The general tendency of women under-reporting sexual partners has been reported by many and elsewhere in Tanzania [40].

Women aged above 20 years who had their first pregnancy at ages younger than 18 years, have a higher HIV prevalence (4.4% CI: 1.20–4.40) compared to those who have their first pregnancy at ages above 18 years (1.5% CI: 0.13–0.72). However, the proportions of early pregnancies of these women are similar to the current teenage pregnancies in the study 12.7% (164/1296). Pregnancies before 18 years of age indicate a younger sexual debut, which has been associated with increased risk of HIV infection [41-43].

Our findings suggest early pregnancies are common in this area at similar proportions in both remote and rural clinics, yet the HIV prevalence had remained relatively low compared to other rural areas in Tanzania [44].

In conclusion, scaling up HIV prevention efforts is mandatory in order to prevent the imminent escalation of the HIV epidemic highly associated with history of STIs, multiple sexual partners and pregnancies at younger age. The observed low level of HIV related knowledge and the fact that few had ever used a condom underscores the urgency for large-scale HIV related programmes. Including remote rural sites as part of routine ANC surveillance is crucial in order to discover imminent silent epidemics such as the one described in this paper. Establishing quality surveillance among pregnant women will allow monitoring of trends and prevent infections to new-borns. Population-based household surveys, behavioural surveillance and relevant social studies would also be necessary. This will allow a wider representation of the risk factors and local context of HIV in the general population (including men and women) to enable customised HIV prevention.

## Competing interests

The author(s) declare that they have no competing interests.

## Authors' contributions

All authors contributed to the paper. All authors conceived the study. The first author conducted the study. B.E. Olsen and M. I. Matee supervised the research. K. I. Yahya-Malima and K Fylkesnes led the analysis. All authors helped to conceptualise ideas, interpret the findings and reviewed drafts of the manuscript.

## Pre-publication history

The pre-publication history for this paper can be accessed here:



## Figures and Tables

**Table 1:** Socio-demographic characteristics among HIV tested antenatal attendees, rural Manyara and Singida regions, Tanzania, 2003–2004.

	**Total n (%)**	**Remote rural n (%)**	**Rural n (%)**
		
**Total**	**1296**	**553 (42.67)**	**743 (57.33)**
**Age groups**			
15–19 years	165 (12.7)	56 (10.1)	109 (14.7)
20–24 years	417 (32.2)	182 (32.9)	235 (31.6)
25–29 years	349 (26.9)	151 (27.3)	198 (26.7)
≥30 years	365 (28.2)	164 (29.7)	201 (27.1)
**Duration of stay**			
> 1 year	1197 (92.4)	534 (96.6)	663 (89.2)
< 1 year	99 (7.6)	19 (3.4)	80 (10.8)
**Marital status**			
Married	1183 (91.3)	525 (94.9)	658 (88.6)
Separated/divorced/widowed/single	113 (8.7)	28 (5.1)	85 (11.4)
**Age when married**			
Never married	93 (7.2)	19 (3.4)	74 (10.0)
< 18 years	203 (15.7)	95 (17.2)	108 (14.5)
≥18 years	1000 (77.1)	439 (79.4)	561 (75.5)
**Religion**			
Christians	999 (77.1)	414 (74.9)	585 (78.7)
Muslims	153 (11.8)	77 (13.9)	76 (10.2)
Others	144 (11.1)	62 (11.2)	82 (11.0)
**Education years**			
None	349 (28.2)	156 (28.2)	193 (26.0)
< 7 years	174 (13.4)	78 (14.1)	96 (12.9)
7 years or more	773 (59.7)	319 (57.7)	454 (61.1)
**Income**			
Yes	204 (15.7)	38 (6.9)	166 (22.3)
No	1087 (83.9)	512 (93.1)	575 (77.7)
**Ever used condom**			
No	1233 (95.1)	532 (96.2)	701 (94.4)
Yes	63 (4.9)	21 (3.8)	42 (5.7)
**Lifetime sexual partners (n)**			
One	1231 (95.0)	525 (94.9)	706 (95.0)
More than one	65 (5.0)	28 (5.1)	37 (5.0)
**Ever VCT**			
No	891 (68.8)	376 (68.0)	515 (69.3)
Yes	405 (31.3)	177 (32.0)	228 (30.7)
Age at first pregnancy*			
< 18 years	158 (14.0)	73 (13.2)	85 (11.4)
≥18 years	974 (86.0)	424 (76.7)	550 (74.0)
**HIV related knowledge**			
No	1153 (88.8)	489 (88.4)	662 (89.1)
Yes	145 (11.2)	64 (11.6)	81 (10.9)

* Analysed for women aged ≥20 years at the time of survey.

**Table 2:** Profiles of antenatal attendees, associated with HIV infection, rural Manyara and Singida within northern Tanzania, 2003–2004.

**Characteristics**	**N (%)**	**HIV prevalence % (95%CI)**	**Crude OR (95%CI)**	**Age-adjusted OR (95%CI)**	**Adjusted for other OR (95%CI)**
**Total**	**1296**	**26 (2.0: 1.34–2.97)**			
**Age groups**					
15–19 years	165 (12.7)	3.03 (1.12–7.30)	1.0	-	1.0
20–24 years	417 (32.2)	1.92 (0.89–3.40)	0.6 (0.20–1.94)		0.7 (0.20–2.47)
25–29 years	349 (26.9)	1.43 (0.53–3.51)	0.5 (0.13–1.63)		0.6 (0.14–2.30)
≥30 years	365 (28.2)	2.19 (1.02–4.44)	0.7 (0.23–2.23)		0.7 (0.20–2.78)
**Residence**					
Remote rural	553 (42.7)	1.3 (0.56–2.71)	1.0	1.0	1.0
Rural	743 (57.3)	2.6 (1.59–4.04)	2.1 (0.85–4.90)	2.0 (0.84–4.87)	2.4 (0.95–6.18)
**Marital status**					
Married	1183 (91.3)	1.9 (1.20–2.85)	1.0	1.0	1.0
Single/divorced/separated	113 (8.7)	3.5 (1.14–9.35)	1.9 (0.65–5.72)	1.8 (0.55–5.57)	1.3 (0.35–4.72)
**Education (years)**					
None	349 (28.2)	3.24 (1.67–5.73)	1.0	1.0	1.0
1–6 years	174 (13.4)	1.15 (0.20–4.52)	0.4 (0.08–1.63)	0.4 (0.08–1.61)	0.3 (0.68–1.64)
7 years or more	773 (59.7)	1.71 (0.96–2.99)	0.5 (0.23–1.19)	0.5 (0.23–1.19)	0.5 (0.20–1.14)
**Number of husbands/sexual partners†**					
1 only	1231 (95.0)	1.7 (1.11–2.69)	1.0	1.0	1.0
> 1	65 (5.0)	7.7 (2.87–17.75)	4.8(1.75–13.17)	5.4(1.91–15.43)	**4.3 (1.42–12.77)**
**Age at first pregnancy ^β^†**					
< 18 years	158 (14.0)	4.4 (1.20–4.40)	1.0	1.0	1.0
≥18 years	974 (86.0)	1.5 (0.71–2.19)	0.3 (0.13–0.72)	0.3(0.12–0.79)	**0.3 (0.13–0.89)**
**Use of contraceptives**					
No	1088 (84.0)	1.6 (0.94–2.54)	1.0	1.0	1.0
Yes	208 (16.0)	4.3 (2.13–8.32)	2.8 (1.25–6.48)	2.9(1.27–6.61)	2.1 (0.79–5.71)
**HIV related knowledge**					
No	1151(88.8)	1.91 (1.11–2.69)	1.0	1.0	1.0
Yes	145 (11.2)	2.76 (0.09–5.43)	1.5 (0.49–4.29)	0.9 (0.91–1.04)	1.8 (0.59–5.56)
**History of genital sore or foul genital discharge†**					
No	1135 (87.6)	1.7 (1.04–2.65)	1.0	1.0	1.0
Yes	161 (12.4)	6.2 (3.19–11.44)	4.6 (2.06–10.39)	4.8(2.14–10.90)	**6.8 (2.78–16.66)**
**Ever used condom**					
No	1233 (95.1)	1.9 (1.15–2.74)	1.0	1.0	1.0
Yes	63 (4.9)	6.4 (2.05–16.25)	3.7 (1.25–11.18)	3.6 (1.18–11.07)	3.2 (0.87–12.06)
**Ever used VCT services**					
No	891 (68.8)	1.3 (0.73–2.41)	1.0	1.0	1.0
Yes	405 (31.2)	3.5 (1.98–5.87)	2.6 (1.20–5.72)	2.6 (1.2–5.72)	(0.72–4.43)

β Analysed for those aged 20 ≥ years at the time of survey,

† Variables included in the final model.

Likelihood-ratio-test (assuming reduced model nested in full model): x^2^(5) = 4.52, P = 0.48

Logistic model for HIV, goodness-of-fit test; x^2^(35) = 31.33, P = 0.64 and predictive power of the model = 78% [45]
